# Combining multiomics to analyze the molecular mechanism of hair follicle cycle change in cashmere goats from Inner Mongolia

**DOI:** 10.3389/fvets.2024.1405355

**Published:** 2024-07-05

**Authors:** Chongyan Zhang, Qing Qin, Zhichen Liu, Yichuan Wang, Mingxi Lan, Dan Zhao, Jingwen Zhang, Zhixin Wang, Jinquan Li, Zhihong Liu

**Affiliations:** ^1^Department of Animal Science, Inner Mongolia Agricultural University, Hohhot, China; ^2^Inner Mongolia Key Laboratory of Sheep & Goat Genetics Breeding and Reproduction, Hohhot, China; ^3^Key Laboratory of Mutton Sheep & Goat Genetics and Breeding, Ministry of Agriculture and Rural Affairs, Hohhot, China; ^4^Northern Agriculture and Livestock Husbandry Technical Innovation Center, Chinese Academy of Agricultural Sciences, Hohhot, China

**Keywords:** apoptosis, ribosomal protein, hair follicle, telogen, anagen, catagen

## Abstract

Sheep body size can directly reflect the growth rates and fattening rates of sheep and is also an important index for measuring the growth performance of meat sheep.Inner Mongolia Cashmere Goat is a local excellent breed of cashmere and meat dual-purpose, which is a typical heterogeneous indumentum. The hair follicles cycle through periods of vigorous growth (anagen), a regression caused by apoptosis (catagen), and relative rest (telogen). At present, it is not clear which genes affect the cycle transformation of hair follicles and unclear how proteins impact the creation and expansion of hair follicles.we using multi-omics joint analysis methodologies to investigated the possible pathways of transformation and apoptosis in goat hair follicles. The results showed that 917,1,187, and 716 proteins were specifically expressed in anagen, catagen andtelogen. The result of gene ontology (GO) annotation showed that differentially expressed proteins (DEPs) are in different growth cycle periods, and enriched GO items are mostly related to the transformation of cells and proteins. The Kyoto Encyclopedia of Genes and Genomes (KEGG) enrichment result indicated that the apoptosis process has a great impact on hair follicle’s growth cycle. The results of the protein interaction network of differential proteins showed that the ribosomal protein family (RPL4, RPL8, RPS16, RPS18, RPS2, RPS27A, RPS3) was the core protein in the network. The results of combined transcriptome and proteomics analysis showed that there were 16,34, and 26 overlapped DEGs and DEPs in the comparison of anagen VS catagen, catagen VS telogen and anagen VS telogen, of which API5 plays an important role in regulating protein and gene expression levels. We focused on API5 and Ribosomal protein and found that API5 affected the apoptosis process of hair follicles, and ribosomal protein was highly expressed in the resting stage of hair follicles. They are both useful as molecular marker candidate genes to study hair follicle growth and apoptosis,and they both have an essential function in the cycle transition process of hair follicles. The results of this study may provide a theoretical basis for further research on the growth and development of hair follicles in Inner Mongolian Cashmere goats.

## 1 Introduction

The integument of cashmere goats is separated into two sections: cashmere and wool. The cashmere production process is periodic and is split into three stages: telogen, anagen, and catagen (1, 2). It has been confirmed that the anagen period is from March to September in a year, which is the process of gradual shedding of cashmere in the previous cycle, new hair follicle regeneration, and cashmere growth; From October to December, the hair follicle development stopped in the catagen, and the cashmere continued to grow, in the later stage, the hair follicle structure began to shrink, the cell apoptosis, and the cashmere growth stopped; From January to March, the cashmere enter the telogen, and the hair follicle structure and cashmere remain static (3). This is a complex process regulated by multiple factors. The biggest feature is that there will be obvious gene expression, cell proliferation, and differentiation. Harris (4) studied that IGF-1 is involved in the periodic regulation of hair follicles, specifically by inducing the proliferation and differentiation of cells such as dermal papilla cells and epithelial cells, thereby regulating the periodic circulation of hair follicles. Yano et al. (5) showed that VEGF also plays a role in promoting hair growth in the periodic cycle of hair follicles. Rendl et al. (6) showed that the expression of BMP2 and BMP4 genes in the secondary hair follicles of cashmere goats in the telogen was higher than that in the growth period, which had the effect of inhibiting the transition of hair follicles from the telogen to the growth period and was one of the important factors regulating the periodic growth of cashmere. Although the cycle process of cashmere growth has been extensively verified. However, the molecular regulation mechanism of cashmere entering three stages has not been revealed.

High-throughput sequencing techniques like RNA-Seq can be used to find novel and low-abundance transcripts, allowing researchers to explore the whole range of gene expression and spot transcript variations between samples (7–9). Proteomics is a potent method for revealing the makeup, distribution, alterations, and interactions of proteins in cells, tissues, or organisms. It includes protein and functional patterns (10). Due to the changes in protein abundance, High-abundance proteins with comparable quality or chemical properties can readily obscure low-abundance proteins (11). Mass spectrometry-based comparative proteomics methods, methods like label-free, iTRAQ, and SWATH allow us to pinpoint proteins that exhibit notable alterations in expression levels on a broad scale under particular circumstances (12–14). Proteomics and other omics together provide more valuable information for investigating the cashmere growth and hair follicle development cycle (15). Proteome and transcriptome are two closely connected downstream and upstream genomics (16, 17). Integrating transcriptome and proteome analysis can provide additional insights not available through conventional individual histology and provide a comprehensive understanding of gene expression and regulation at every stage (18).

There, we examined the cashmere growth fluctuations in cashmere goats using the transcriptome and proteome for the first time. We additionally investigated the impact of various periods on cashmere development, transcriptional alterations, and protein levels. Our results fill a gap in the study of cashmere growth and hair follicle development.

## 2 Materials and methods

### 2.1 Animal welfare disclaimer

The Yiwei White cashmere Goat Breeding Farm in Erdos, Inner Mongolia, provided grazing conditions for the Inner Mongolian cashmere goats used in this experiment. The Inner Mongolia Agricultural University’s experimental animal management committee has authorized every experimental technique used in this work. The present investigation involved the collection of skin samples by the International Guiding Principles for Biomedical Research involving animals. The experiment was approved by the Inner Mongolia Agricultural University’s Special Committee on Scientific Research and Academic Ethics, which is in charge of approving the university’s biomedical research ethics [Approval No: (2020)056, project title: the International Guiding Principles for Biomedical Research involving animals, approval date: May 6th, 2020]. Three mature cashmere goats at a time, with similar growth, age, and feeding circumstances, serve as samples for three periods. The RNA of the samples is then mixed. Nine cashmere goats from the same family had skin samples taken. In the Department of Surgery, samples were taken from the middle of the scapula at a length of 10–15 cm. The experimental animals were not sacrificed, and after the skin was sampled, they received medication treatments that did not interfere with their normal growth. The 3-cm-diameter skin samples were collected, immediately rinsed with PBS, and quickly frozen in liquid nitrogen. After being transported to the lab in a liquid nitrogen tank, the samples were kept in a freezer at −80°C.

### 2.2 Protein extraction and digestion

The lysis process involved pulverizing nine skin samples in liquid nitrogen, adding the powder to lysis buffer (8-mg urea and 1% protease inhibitor), then lysing the mixture using ultrasound. The supernatant was then transported to a fresh centrifuge tube after the cell debris was eliminated using a centrifuge set at 12,000 g for 10 min at 4°C. Following the collection of the filtrate, the processed samples were held at −80°C while protein quantification was carried out using the BCA ProteinAssayKit (Abcan, China).

### 2.3 Mass spectrometry for label-free LC/MS

Peptide data were obtained using information-dependent capture (IDA) and sequential window acquisition of all theoretical spectra-mass spectrometry (SWATH-MS) in the Sciex LC–MS/MS system (Framingham, MA, United States). A C18 column measuring 75 μm by 15 cm was used to inject about 2 μg of polypeptide for separation. A linear gradient of 0.1% formic acid in acetonitrile and 0.1% formic acid in water was used to separate the peptides (120 min, 500 nL/min, from 5 to 80%). The IDA parameters were as follows: automated collision energy; 350–1,800 m/z for time-of-flight mass spectrometry collection; 400–1,800 m/z for MS/MS and IDA scan. The nominal resolution was set at 30,000. The following were the SWATH-MS conditions: nominal resolutions of MS1 and MS2, 30,000 and 15,000, respectively; 150–1,200 m/z, MS1 mass range; 100–1,500 m/z, MS2 mass range.

### 2.4 Label-free LC/MS quantitative and qualitative and profiling

Peptide identification was performed using the UniProt/SWISS-PROT/*Capra hircus* database^1^ and Protein Pilot v4.5 software (Sciex, Framingham, MA, United States). A 1% false discovery rate (FDR) was used to filter the results. Trypsin was chosen as the enzyme and two missed cleavage sites were permitted among the search parameters. There was a 15 ppm peptide mass tolerance and a 20 mmu fragment mass tolerance. The data were imported into the software PeakView v2.1 (Sciex, Framingham, MA, United States), and the SWATH database was searched using the ion library produced by Protein Pilot. PeakView produced the extracted ion chromatograms (XICs) by processing the target and nontarget data. The findings were then explained and subjected to a quantitative analysis using the MarkerView v3.0 program (Sciex). With MarkerView, one may quickly examine data to identify the proteins that are differentially expressed (DEPs). The fold change analysis and t-tests were merged into principal component analysis (PCA) and volcano plot analysis. DEPs were identified using a fold change >2 or fold change <0.5, as well as statistical significance (*p* < 0.05).

### 2.5 Total RNA extraction from skin and construction of sequencing library

Using an RNAiso Plus Kit (TRIzol technique), total RNA was extracted from the skin of three cashmere (one period) goats. The RNA samples from three goats were combined after the total RNA was examined for purity and integrity using a sterile UV–vis spectrophotometer and an Agilent 2,100 bioanalyzer, respectively. RNA samples were collected in September (anagen), December (catagen), and March (telogen) using the same method and operation. The whole RNA was kept at −80°C in a freezer.

The Illumina TruSeqTM RNA Sample Preparation Kit’s operating instructions were followed while creating the cDNA library for transcriptome sequencing. By combining equal parts of each, the total RNA from the three cashmere goats was combined. After the mRNA was separated into 100–400 bp mRNA, it was purified using oligo-dT magnetic beads. Exonucleases, polymerases, and fragmented mRNA were used to create double-stranded cDNA. Using a Bio-Rad Certified Low-Range Ultra Agarose Kit, the ends of the double-stranded cDNA fragments were blunted, and the double-stranded cDNAs were phosphorylated to ligate the sequencing adapters and poly (A) tail. The sizes of the cDNA recovered were determined to be 200–300 bp. A sequencing library was created by PCR amplification of cDNA, and a TBS-380 device was used for library quality verification. An Illumina HiSeq^TM^ 2000 sequencing platform was used to perform paired-end sequencing of the cDNA. The samples were sequenced by Beijing Baimaike Biotechnology Co., Ltd. using an A2 × 100 bp sequencing test.

### 2.6 Bioinformatics analysis

Using the *Capra hircus* genome annotation as background and the David database^2^ with default parameters, GO functional and KEGG pathway annotation was carried out. Obtained, encompassing analyses of the cellular component (CC), molecular function (MF), and biological process (BP).

The Search Tool for the Retrieval of Interacting Genes/Proteins (STRING)^3^ database was used to perform the Protein–protein Interaction (PPI) study of the differentially expressed proteins between goat breeds, as previously mentioned. A minimum needed interaction score of >0.4 was used in the construction of the network. The network was visualized using Cytoscape v.3.9.0 software (Cytoscape Consortium, San Diego, CA, United States). Additionally, the hub proteins of the PPI network were investigated using the McCreight (MCC) approach with the help of the Cytoscape add-on CytoHubba.

### 2.7 Parallel reaction monitoring validation for differentially expressed proteins

The protein abundances were examined using the parallel reaction monitoring (PRM) approach to verify the accuracy of the SWATH-based proteomic data. The peptides were separated using a liquid chromatography-tandem quadrupole mass spectrometry system and dissolved in liquid chromatography mobile phase A (0.1% formic acid solution). The final step was processing the generated MS data with Skyline (64-bit, 22.2.0.351) (19).

### 2.8 Quantitative real-time PCR

For quantitative reverse transcription PCR (qPCR) analysis, the cDNA that was previously acquired was utilized. Primer 3.0 software was utilized to create the gene-specific primers for q-PCR, and Sangon Biotech Co., Ltd. (Shanghai, China) synthesized them. Table 1 lists the fragment sizes and primer sequences. For the q-PCR, a 20-μL reaction volume was used together with a PrimeScript RT Reagent Kit (TaKaRa, Beijing), 10 μL of 2 × SYBR Premix Ex Taq II (TaKaRa), 2 μL of cDNA, and 0.5 μL of each primer. A Bio-Rad IQ5 multicolor real-time PCR detection system (Hercules, CA, United States) was used to evaluate the reaction. The reference used was the β-actin. The 2 − ΔΔCT method was used for the qRT-PCR study, and SPSS software (version 17.0) was used for the statistical analysis. The format for values is mean ± standard deviation.

**Table 1:** Primer sequence information of differentially expressed genes.

Primer name	Primer sequence (5′–3′)	Product length/bp
VEGFA	F: TACCACCACCACCACCACCATC R: CACCCGCCCATGAATGCTTCTG	133
RAC1	F: CACTGTCCCAACACACCCATCATC R: GGCGTCAGCTTCTTCTCCTTCAG	98
NOTCH1	F: GCAAGTGCATCAACACGCTG R: TGGAACTCCCCGATCTGGTC	145
LHX2	F: CTACTACAATGGCGTGGGCA R: ACTTCATGGTCCGAAGCTGG	212
KRT2	F: TCTGCAGCCTCTCAATGTGA R: GGTGCCCACATCGATTTGTT	184
FGF21	F: TGCTGGCTGTCCTCCTGCTAG R: TCCTGGGCATCATCCGTGTAGAG	118bp

## 3 Results

### 3.1 Identification of proteins in goat skin

**Figure 1:**
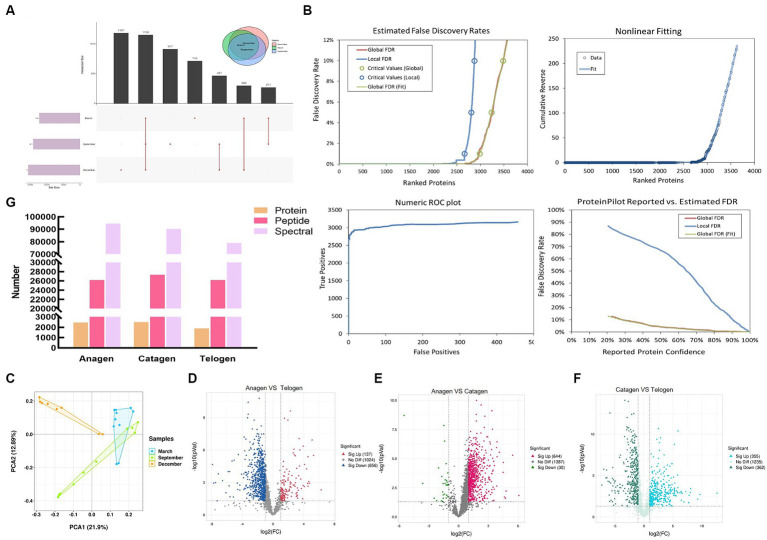
Identification of proteins of skins from goats. **(A)** UpSet plots depicting the number of unique and shared expressed proteins between different periods. **(B)** Estimated False Discovery Rates, Nonlinear Fitting of, Numeric ROC plot, and ProteinPilot Reported vs. Estimated FDR of all protein. **(C)** Two-dimensional scatter plot of quantitative principal component analysis of protein among samples. **(D)** Volcano map of differentially expressed proteins in anagen and telogen. **(E)** Volcano map of differentially expressed proteins in anagen and catagen. **(F)** Volcano map of differentially expressed proteins in catagen and telogen.

Cashmere goat skin proteome analysis was carried out in three stages using the constructed proteome map liquid chromatography–tandem mass spectrometry approach to examine changes in protein dynamics during the growth of goat wool. The proteome and transcriptome profiling of inner mongolia cashmere goat skin with anagen (Sept), catagen (Dec), and telogen (March) were constructed. Totally, with a false discovery rate (FDR) ≤ 0.01, 1890 proteins and 20,116 distinct peptides were identified in March; 2,460 proteins and 26,191 distinct peptides were identified in September; 2,505 proteins and 27,352 distinct peptides were identified in December. 716, 917, and 1,187 proteins were specifically expressed in March, September, and December (Figure 1A). Among three groups, a total of 2,983 proteins and 38,569 distinct peptides were identified (Figure 1B). We used principal component analysis (PCA) to cluster the protein to evaluate the quantitative consistency of the proteomics data. (Figure 1C). The PCA result showed that most of the samples in the same period were clustered together and crossed in the quadrants of different periods, indicating that proteins were related to different periods of hair follicle development. According to Figure 1G, it can be found that the number of peptides and maps did not change significantly during the transition from the hair follicle growth period to the degenerative period, that is, the basic composition of these proteins did not change. However, in this process, the number of proteins has changed significantly, and the change rule is that the number of proteins in the growth period-degeneration period-rest period changes from more to less and then to more. The number of proteins gradually increased during the growth period to the degenerative period, revealing the important role of these proteins in the development of hair follicles and the growth of cashmere. However, there are two primary phases to the growth of hair follicles, the first is the growth of primary hair follicles, and the second is the creation of cashmere fiber. Different proteins are involved in these two processes, but the total protein number is gradually increasing. Previous studies have shown evidence for the presence of molecular spatial gradients during the formation of hair follicles. The hair follicle structure as a function of the spatial dimension was then examined in our IDA dataset to capture this at the proteome level. The study used IDA data with fold changes ≥2 or < 0.05 and a *p*-value <0.05 to identify 137 proteins that were found to be highly expressed in anagen compared with telogen, while 656 proteins were highly expressed in telogen compared with anagen (Figure 1D). In anagen *VS* catagen, 644 up-regulated proteins and 30 down-regulated proteins were identified (Figure 1E). In catagen *VS* telogen 355 up-regulated proteins and 362 down-regulated proteins were identified (Figure 1F). These proteins play different roles in three stages to promote hair follicle growth and apoptosis.

### 3.2 Functional enrichment analysis of differentially expressed proteins

**Figure 2:**
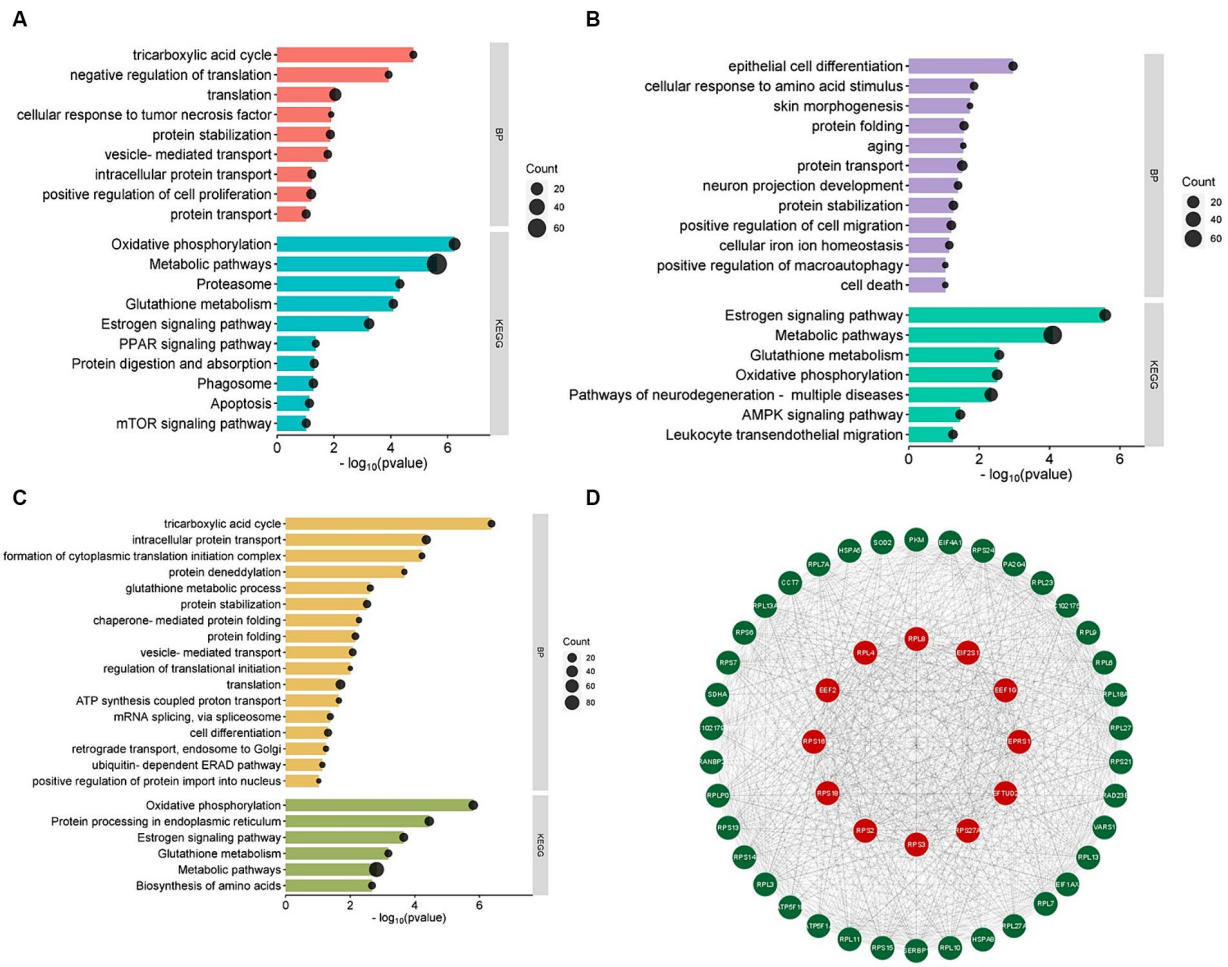
Differentially expressed protein analysis. **(A)** GO function and KEGG pathway analysis of differentially expressed proteins in anagen and catagen. **(B)** GO function and KEGG pathway analysis of differentially expressed proteins in catagen and telogen. **(C)** GO function and KEGG pathway analysis of differentially expressed proteins in telogen and anagen. **(D)** Protein–protein interaction regulatory network.

To explore the potential functions of up-regulated and down-regulated Differentially Expressed Proteins (DEPs) identified in this study, GO and KEGG enrichment analysis was performed on DEPs. The results of GO enrichment in three different periods showed that all categories were enriched, and the biological process items were mainly enriched. The main GO terms of biological processes in the anagen *VS* catagen groups include translation, positive regulation of cell proliferation, protein stabilization, intracellular protein transport, protein transport, vesicle-mediated transport, etc. (Figure 2A). It shows that 674 differential proteins play an important role in promoting cashmere growth by hair follicles. In the catagen *VS* telogen comparison group, protein transport, skin morphogenesis, aging, cell death, positive regulation of macroautophagy, etc. were enriched (Figure 2B), indicating that 717 differential proteins were mainly used in hair follicle apoptosis. In the telogen *VS* anagen comparison group, translation, intracellular protein transport, cell differentiation, ubiquitin-dependent ERAD pathway, regulation of translational initiation, positive regulation of protein import into the nucleus etc. were enriched (Figure 2C), it is indicated that the role of the up-regulated protein in the transition from telogen to anagen is hair follicle apoptosis, while the role of the down-regulated protein is the growth or maintenance of cashmere.

We performed KEGG enrichment analysis on all DEPs. Within these three divisions, DEPs were mainly enriched in Metabolic pathways, Oxidative phosphorylation, and Estrogen signaling pathways. For the anagen *VS* catagen group, DEPs were significantly enriched in Phagosome, Proteasome, Protein digestion and absorption, Apoptosis, mTOR signaling pathway, and PPAR signaling pathway (Figure 2A). For the catagen *VS* telogen group, DEPs were significantly enriched in Pathways of neurodegeneration-multiple diseases, AMPK signaling pathway, Glutathione metabolism, and Leukocyte transendothelial migration (Figure 2B). For the telogen *VS* anagen group, Protein processing in the endoplasmic reticulum, Glutathione metabolism, Biosynthesis of amino acids were significantly enriched (Figure 2C). From the results of different pathways enriched from different periods, it is further verified that different proteins are enriched and functioned at different periods to maintain the cycle of cashmere and the cycle of cashmere growth.

Protein–protein interaction (PPI) network utilizing STRING was created to ascertain the relationships among these DEPs. The PPI network of DEPs contains 11,621 edges and 1,064 nodes. Additionally, the top 50 proteins in the network were obtained based on the analysis using Cytoscape Degree (Figure 2D). Ribosomal protein family (RPL4, RPL8, RPS16, RPS18, RPS2, RPS27A, RPS3), Elongation Factor family (EEF1G, EEF2, EFTUD2), Translation Initiation Factor family (EIF2S1) and Glutamyl-prolyl-tRNA synthetase 1 (EPRS1) interacted strongly with each other. In addition, we found these proteins were significantly enriched in translation, cell differentiation, positive regulation of cell proliferation, negative regulation of translation, etc. based on PPI and function enrichment results. We speculated that the ribosomal protein might be involved in development regulation, cell differentiation, and apoptotic in hair follicles of cashmere goats at different stages.

### 3.3 Analysis of differences in gene expression

To explore the molecular mechanism of initiating periodic changes in hair follicles at the genomic level. Three skin samples from cashmere goats aged 3, 9, and 12 months were subjected to transcriptome sequencing analysis using the Illumina sequencing technology. The raw read count that was retrieved was 173,925,724. Over 94% of bases with a mass value greater than 30 (and an error rate of less than 0.1%) were found in all sequenced samples. 169,651,142 clean readings were obtained thereafter data filtering. The retrieved 220,696,075 mapped reads had a comparability rate of 92.58–93.22% with the reference genome of cashmere goats (Table 2).

**Table 2:** The mapping of clean data.

Library	Raw reads	Clean reads	GC (%)	N (%)	Q30 (%)
March	83,697,110	81,577,244	53.07	0.0111	94.54
September	82,490,126	80,504,704	53.28	0.0113	94.57
December	7,738,488	7,569,194	52.98	0.0112	94.5

**Figure 3:**
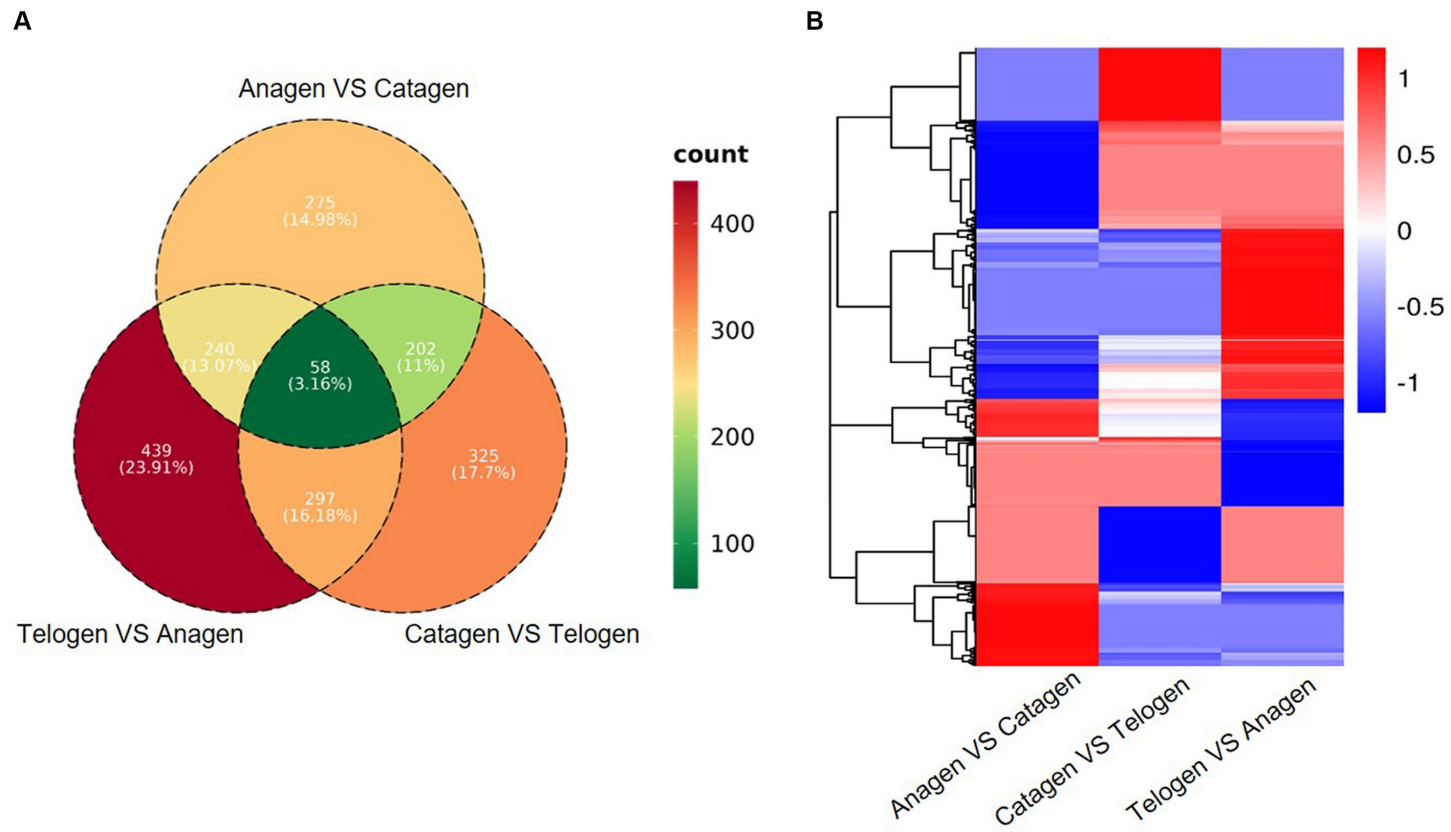
**(A)** Venn diagram of differentially expressed genes in Zhongwei goat skin at three developmental stages. **(B)** Cluster analysis of gene expression in three groups.

By comparing the levels of gene expression in each sample, Differentially Expressed Genes (DEGs) were identified. Totally, 775, 882, and 1,034 DEGs were determined in comparison of anagen *VS* catagen, catagen *VS* telogen, and telogen *VS* anagen (q ≤ 0.05, |FC| > 2). Furthermore, 58 DEGs that overlapped were found in each of the three comparison groups (Figure 3A). Cluster analysis of DEGs found that most of the DEGs in the anagen were down-regulated, most of the DEGs in the telogen were up-regulated, and the DEGs in the catagen period were in between (Figure 3B).

### 3.4 Integrated analysis of transcriptome and proteome

**Figure 4:**
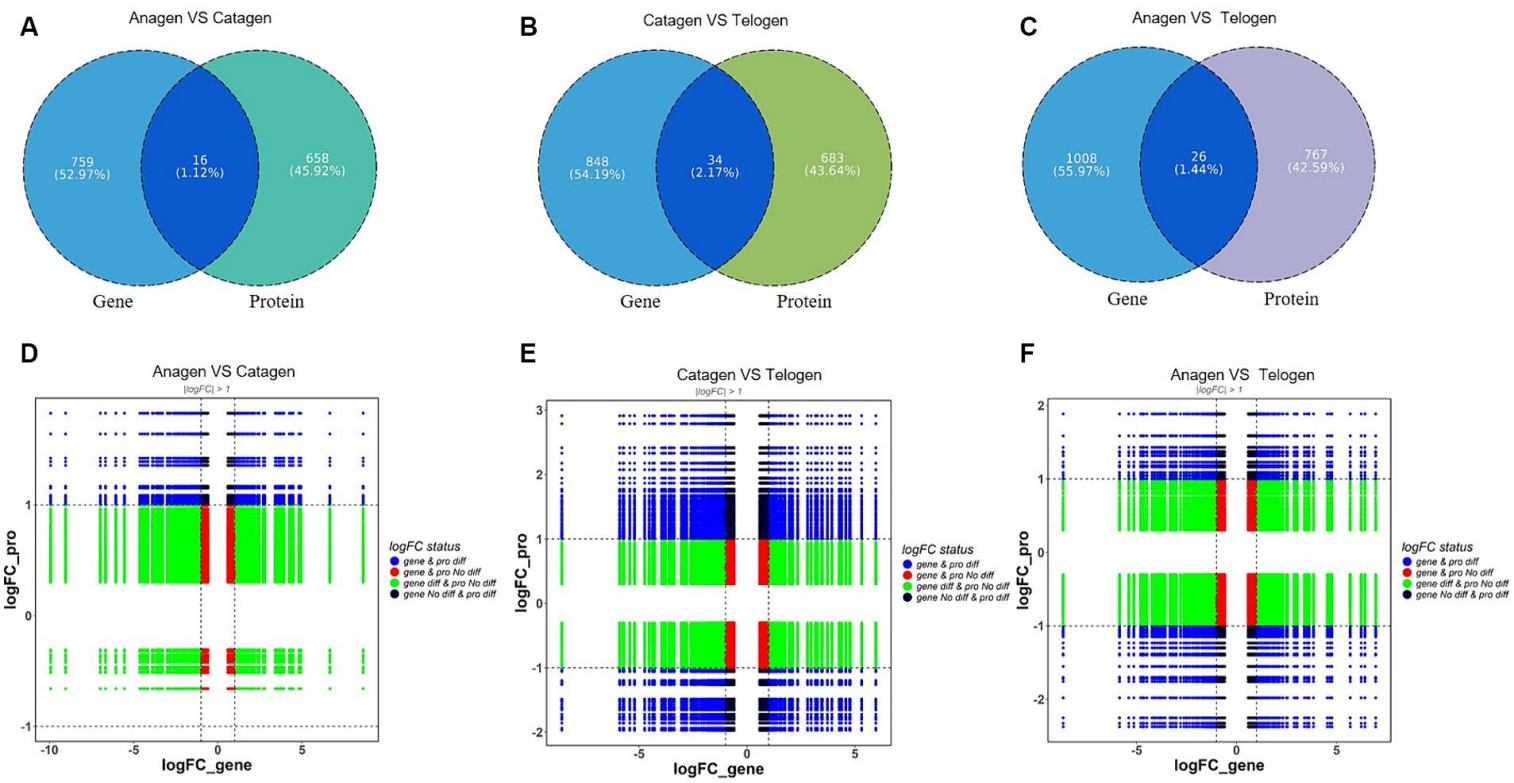
Analysis of differentially expressed genes between transcriptome and proteome. **(A)** Proteome and transcriptome different expression Venn diagrams of anagen and catagen. **(B)** Proteome and transcriptome different expression Venn diagrams of catagen and telogen. **(C)** Proteome and transcriptome different expression Venn diagrams of telogen and anagen. **(D)** Nine-quadrant diagram of differentially expressed genes between transcriptome and proteome in anagen and catagen. **(E)** Nine-quadrant diagram of differentially expressed genes between transcriptome and proteome in catagen and telogen. **(F)** Nine-quadrant diagram of differentially expressed genes between transcriptome and proteome in telogen and anagen.

The DEPs and DEGs were compared within each group to assess the correlation between the transcriptome and proteome data. Three clusters of genes with 16, 34, and 26 overlapped copies were found (Figures 4A–C).

Translational regulatory mechanisms explain the correlation between transcriptome and proteome data. At this point, nine-quadrant association analyses can be very helpful. Among the 16 genes/proteins expressed in anagen *VS* catagen, TCHH was enriched in the 1 quadrant; in the 2 quadrants, COL2A1 is enriched; in the 4 quadrant, *API5*, *CDSN*, *FBN1*, *TPPP3*, *UCHL3* were enriched. *HSPA8*, *LDHB*, *OGN*, *PON3*, *RPS24*, *SBSN*, and *SOAT1* were enriched in the 5 quadrants. In the 6 quadrants, *GNG12* and *CCT2* were enriched (Figure 4D). Of the 34 genes/proteins expressed in catagen *VS* telogen, *COCH* was enriched in the 1 quadrant; *APOA1*, *COL1A2*, *EIF4A2*, *MYH11*, and *RPL27A* were enriched in the 2 quadrant; *ALDOC*, *API5*, *KRT10*, *SLC25A24*, *TGFBI*, and *UCHL3* were enriched in the 4 quadrants; *FBLN5*, *LDHBC4*, *AHCY*, *DCN*, *DNAJA2*, *DPT*, *FBN1*, *GANAB*, *LUM*, *MMP2*, *PADI3*, *QSOX1*, *SLC1A5*, and *TES* were enriched in the 5 quadrants; *AdipoQ*, *GNG12*, *PLIN3*, *PRDX4*, and *PSMC4* were enriched in the 6 quadrants; *PCP4L1* and *CDSN* were enriched in the 7 and 8 quadrants (Figure 4E); The 26 genes/ proteins expressed in telogen *VS* anagen, *RPL23* was enriched in the 2 quadrantss; *DNAJA2, DPT, GPX3, PCP4L1, RPL21, RPL22,* and *SLC25A24* were enriched in the 4 quadrant; *ACTG2, COCH, EIF4A2, GLUL, GSR, GSS, HSPH1, MAOA, PMM2, RPL10, RPL4, TPM2, TPPP3, TXN2,* and *UCHL3* were enriched in the 5 quadrant; API5 an lation-level regulation may be the reason why certain proteins abundant in the sixth, eighth, and ninth quadrants had higher abundances than RNA, whereas some proteins in the first, second, and fourth quadrants had lower abundances than the comparable RNAs. From the anagen-catagen and the catagen-telogen, both are expressed by API5; the high expression of RPL family members was found in the process of telogen-anagen.

### 3.5 Key protein structure domain analysis

**Figure 5:**
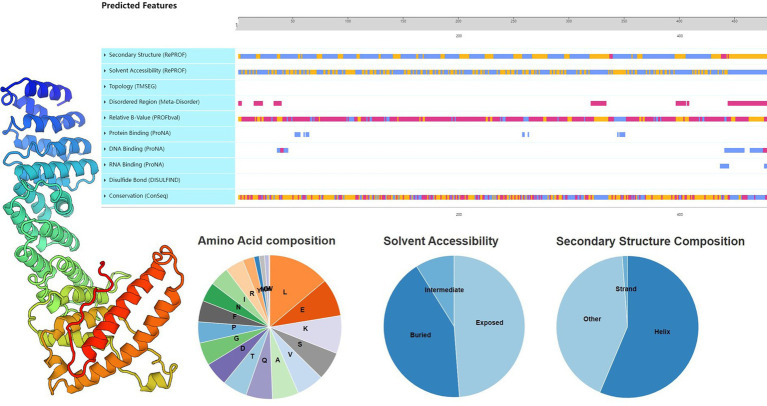
Structural analysis of key proteins. Primary, secondary, and tertiary structure analysis of API5 protein.

The main function of the gene is to store and transmit genetic information, through the process of transcription and translation, and ultimately the formation of protein, and then play a role in the body. Proteins account for 50% of the dry mass of cells and play a role in everything the organism does, they are all composed of the same 20 amino acids. The API5 sequence length of 504, 56.1% is Helix, 1.2% is Strand, and 42.7% is non-helical and non-folded random structure. On Solvent Accessibility,42.1% of the area is Buried and 48.7% is Exposed (Figure 5).

### 3.6 PRM quantitative results

To confirm the correctness of the proteomic data in three comparison groups, three proteins were chosen for PRM quantification based on the expression level of the DEPs. The findings of the SWATH data analysis and PRM detection ratio showed a steady general trend, suggesting that the proteomic data were dependable and repeatable (Table 3).

**Table 3:** PRM result compared with SWATH-based quantitative result.

Gene name	Peptide	Telogen expression (PRM)	Telogen expression (SWATH)	Anagen expression (PRM)	Anagen expression (SWATH)	Catagen expression (PRM)	Catagen expression (SWATH)
TPM4	RFEKPLEEK	4.57E+04	18.12	9.93E+04	22	3.31E+05	31.86
SNX2	VSHYIINSLPNRRFK	2.34E+03	4.11	2.48E+05	10.84	7.41E+03	4.29
PSMA1	IGGAQNRSYSK	2.15E+05	8.2	2.85E+05	11.7	2.44E+04	6.17

### 3.7 Quantitative real-time PCR validation

**Figure 6:**
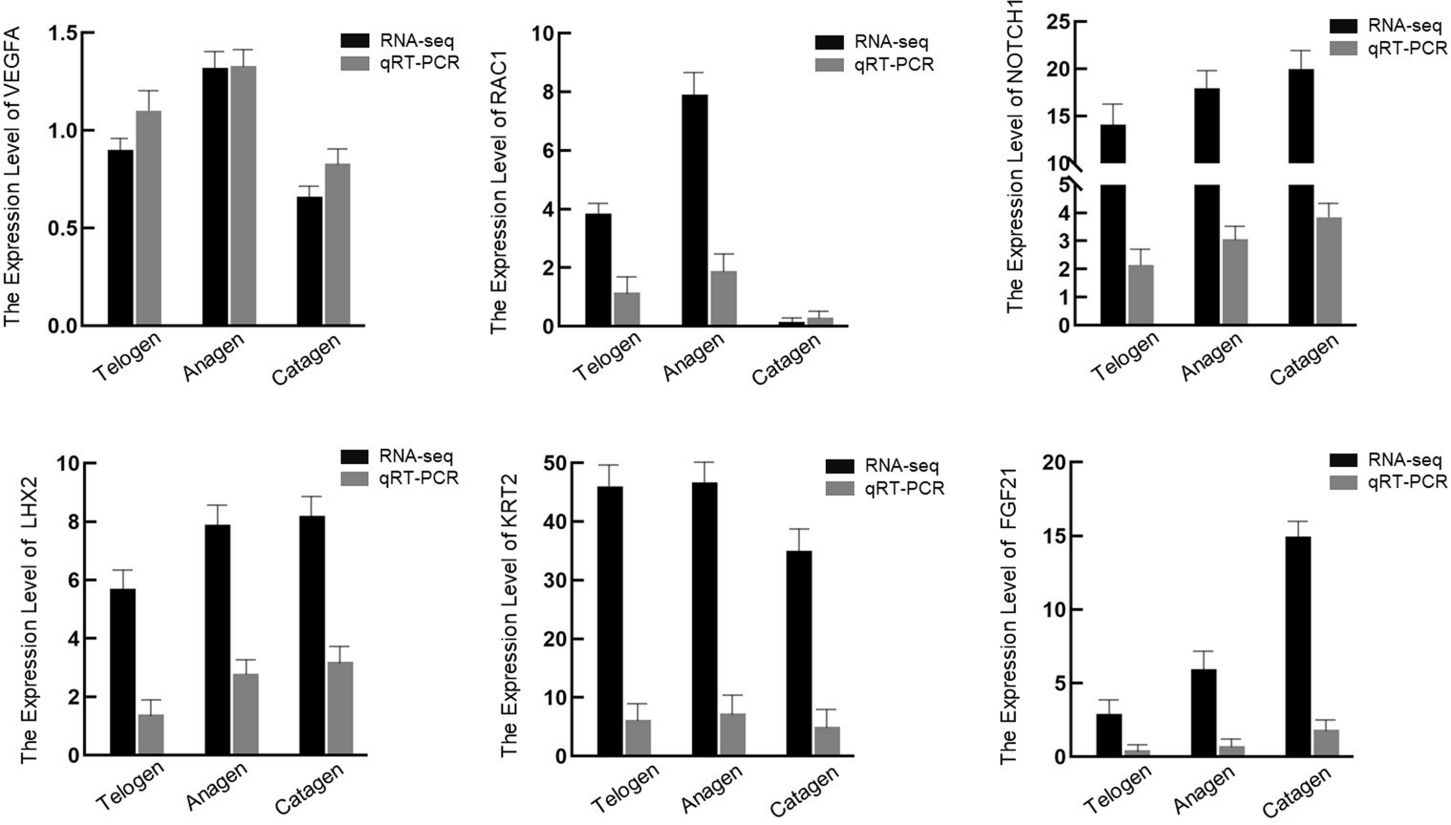
mRNA expression levels for six genes in the skin samples of different period cashmere goats examined, via quantitative polymerase chain reaction, to verify the RNA sequencing data.

Six DEGs were chosen at random to verify the correctness of the transcriptome data (Figure 6). Since the transcriptome results matched the DEGs’ real-time fluorescence expression patterns, our transcriptomic data were considered reliable.

## 4 Discussion

From phenotypic to molecular processes, researchers have attempted to investigate the periodic changes in cashmere goat hair follicles since the 1960s. Dynamic changes in gene regulation and protein expression occur during this process (20). Since gene expression at the RNA and protein levels varies with time and tissue, transcriptome sequencing provides a direct means of investigating changes in gene expression, while proteome identification provides a direct means of investigating changes in protein function (21). We used multi-omics technology to jointly analyze the cycle conversion mechanism of cashmere goat hair follicles.

Firstly, the protein expression at different stages was identified. As the hair follicle began to grow and develop to the hatching hair grew out of the body surface and then to the hair follicle atrophy and apoptosis, the number of proteins was consistent with its changes. We speculate that there are specific proteins that function at different stages and promote the growth and apoptosis of hair follicles. The differential proteins were analyzed, and the enriched functional results including cell proliferation, protein transport cell death, etc., were as we speculated. Secondly, we performed gene mining on skin samples at different stages through transcriptomics. It was found that, like proteomics, the number of gene expressions fluctuated with the growth and apoptosis of hair follicles, and specific expression genes appeared at different stages. Among the 2,691 DEGs identified, 76 DEGs were overlapped with DEPs. Subsequently, we focused on the genes/proteins of 76 and finally locked the Nuclear protein Apoptosis Inhibitor 5 and ribosomal protein (RP) through correlation analysis.

Nuclear protein Apoptosis Inhibitor 5 (API5) prevents cells from going through the apoptotic process. This protein was first discovered in serum-deprived cells that survived, and it was subsequently discovered to be overexpressed in some malignancies and to control apoptosis in both vertebrates and invertebrates (22–24). In our study, we found that the API5 protein is regulated by post-transcriptional and translational levels, and the target gene inhibits its translation during the anagen-catagen and catagen-telogen periods. In these two stages, hair follicle cells undergo a process from initial apoptosis to complete apoptosis, and the expression of API5 shows a continuous downward trend, which is consistent with the cycle transition of goat hair follicles. During the telogen-anagen period, the API5 protein is regulated by post-transcriptional and translation levels, and the target gene promotes its translation. At this stage, hair follicles begin to grow and develop, API5 functions to inhibit apoptosis, and API5 expression increases; Numerous studies have demonstrated that API5 is up-regulated in human cancers of the cervix, prostate, lung, colon, and other related tissues in mouse fibroblasts, and has significant anti-apoptotic ability (22, 24–26). However, there is no relevant report on the skin cycle of goats. In our study, API5 protein showed a trend of increasing first and decreasing in the process of telogen-growth-degeneration, and the gene expression pattern was the same trend. For the first time, we discovered that in goats, API5 is connected to both apoptosis and hair follicle growth. Combined with functional analysis, API5 was enriched in Apoptosis in the biological process, which further confirmed the important role of API5 in the expression of hair follicle cycle transition in goats. Therefore, API5 will also be the focus of the study of the skin cycle.

Ribosomal protein (RP) is a general term for all proteins involved in the formation of ribosomes. It has a significant role in intracellular protein biosynthesis and has been found in many organisms. It is expressed in different organs and tissues of the human body. Currently, eukaryotic cells contain about 80 different types of ribosomal protein (RP). These RPs are referred to as ribosomal protein large (RPL) and ribosomal protein small (RPS) based on where they originate from—big and small subunits, respectively. A variety of studies have found that RP not only has the function of protein translation but also participates in the regulation of DNA replication, transcription, repair, RNA splicing, modification, cell proliferation, apoptosis, and other functions (27–29). In this study, RPL family members are the most connected proteins in the protein interaction network. In the co-expression module of DEGs and DEPs, they are highly expressed in the telogen-growth period. This may mean that the ribosomal protein caused a significant shift in the internal molecular microstate of the epidermis when the hair follicle transitioned from the resting phase to the new round of cycle growth phase.

According to our research, ribosomal protein and apoptosis inhibitor 5 had an impact on the growth and development of hair follicles in Inner Mongolian Cashmere goats. This will support our ongoing research into the mechanisms behind the proliferation and death of hair follicles in Inner Mongolian cashmere goats.

## 5 Conclusion

We provide comprehensive proteomic and transcriptomic data for inner mongolia cashmere goat skin with different three development stages. The gene expression at different stages and the function of protein volatilization were systematically studied. The data revealed the API5 may be involved in hair follicle apoptosis and influence the process of hair follicle cycle transition, and the transition of hair follicles from telogen to anagen is facilitated by the high expression of the RPL family, which also stimulates hair growth. The results provide a useful transcriptomic and proteomic resource and a broad understanding of the proliferation apoptosis mechanism underlying hair follicles.

## Data availability statement

The data presented in the study are deposited in the ProteomeXchange Consortium repository, accession number PXD036685.

## Ethics statement

Samples were collected by the Guidelines for Experimental Animals of the Ministry of Science and Technology (Beijing, China) and were approved by the experimental animal ethics committee of Inner Mongolia Agricultural University (GB 14925–2001).

## Author contributions

CZ: Writing – original draft, Writing – review & editing. QQ: Writing – review & editing. ZhicL: Writing – review & editing. YW: Writing – review & editing. ML: Writing – review & editing. DZ: Writing – review & editing. JZ: Writing – review & editing. ZW: Writing – review & editing. JL: Writing – review & editing. ZhihL: Writing – review & editing.

## Footnotes

^1^ https://www.UniProt.org/#

^2^ https://david.ncifcrf.gov/

^3^ https://cn.string-db.org/

## References

[1] Ge W Wang SH Sun B Zhang YL Shen W Khatib H. Melatonin promotes cashmere goat (*Capra hircus*) secondary hair follicle growth: a view from integrated analysis of long non-coding and coding RNAs. Cell Cycle. (2018) 17:1255–67. doi: 10.1080/15384101.2018.1471318, PMID: 29895193 PMC6110581

[2] Geng R Yuan C Chen Y. Exploring differentially expressed genes by RNA-seq in cashmere goat (*Capra hircus*) skin during hair follicle development and cycling. PLoS One. (2013) 8:e62704. doi: 10.1371/journal.pone.0062704, PMID: 23638136 PMC3640091

[3] Liu Z Yang F Zhao M Ma L Li H Xie Y. The intragenic mRNA-microRNA regulatory network during Telogen-Anagen hair follicle transition in the cashmere goat. Sci Rep. (2018) 8:14227. doi: 10.1038/s41598-018-31986-2, PMID: 30242252 PMC6155037

[4] Harris PM McBride BW Gurnsey MP Sinclair BR Lee J. Direct infusion of a variant of insulin-like growth factor-I into the skin of sheep and effects on local blood flow, amino acid utilization and cell replication. J Endocrinol. (1993) 139:463–72. doi: 10.1677/joe.0.1390463, PMID: 8133213

[5] Yano K Brown LF Detmar M. Control of hair growth and follicle size by Vegf-mediated angiogenesis. J Clin Invest. (2001) 107:409–17. doi: 10.1172/jci11317, PMID: 11181640 PMC199257

[6] Rendl M Polak L Fuchs E. Bmp signaling in dermal papilla cells is required for their hair follicle-inductive properties. Genes Dev. (2008) 22:543–57. doi: 10.1101/gad.1614408, PMID: 18281466 PMC2238674

[7] Wang L Cai B Zhou S Zhu H Qu L Wang X. RNA-seq reveals transcriptome changes in goats following myostatin gene knockout. PLoS One. (2017) 12:e0187966. doi: 10.1371/journal.pone.0187966, PMID: 29228005 PMC5724853

[8] Wang Z Gerstein M Snyder M. RNA-seq: a revolutionary tool for Transcriptomics. Nat Rev Genet. (2009) 10:57–63. doi: 10.1038/nrg2484, PMID: 19015660 PMC2949280

[9] Ozsolak F Milos PM. RNA sequencing: advances, challenges and opportunities. Nat Rev Genet. (2011) 12:87–98. doi: 10.1038/nrg2934, PMID: 21191423 PMC3031867

[10] Pandey A Mann M. Proteomics to study genes and genomes. Nature. (2000) 405:837–46. doi: 10.1038/35015709 10866210

[11] Wilkins MR Sanchez JC Gooley AA Appel RD Humphery-Smith I Hochstrasser DF. Progress with proteome projects: why all proteins expressed by a genome should be identified and how to do it. Biotechnol Genet Eng Rev. (1996) 13:19–50. doi: 10.1080/02648725.1996.10647923 8948108

[12] Marcelino I Almeida AM Brito C Meyer DF Barreto M Sheikboudou C. Proteomic analyses of *Ehrlichia ruminantium* highlight differential expression of Map1-family proteins. Vet Microbiol. (2012) 156:305. doi: 10.1016/j.vetmic.2011.11.022 22204792

[13] Ouyang H Wang Z Chen X Yu J Li Z Nie Q. Proteomic analysis of chicken skeletal muscle during embryonic development. Front Physiol. (2017) 8:281. doi: 10.3389/fphys.2017.00281, PMID: 28533755 PMC5420592

[14] Murgiano L D'Alessandro A Egidi MG Crisà A Prosperini G Timperio AM. Proteomics and Transcriptomics investigation on longissimus muscles in large white and Casertana pig breeds. J Proteome Res. (2010) 9:6450–66. doi: 10.1021/pr100693h, PMID: 20968299

[15] Bourmaud A Gallien S Domon B. Parallel reaction monitoring using quadrupole-orbitrap mass spectrometer: principle and applications. Proteomics. (2016) 16:2146–59. doi: 10.1002/pmic.201500543, PMID: 27145088

[16] Hu Y Zhang Y Šmarda P Bureš P Guo Q. Transcriptome and proteome associated analysis of flavonoid metabolism in haploid *Ginkgo biloba*. Int J Biol Macromol. (2023) 224:306–18. doi: 10.1016/j.ijbiomac.2022.10.125, PMID: 36257359

[17] Zhao J Yang J Xie Y. Improvement strategies for the Oral bioavailability of poorly water-soluble flavonoids: an overview. Int J Pharm. (2019) 570:118642. doi: 10.1016/j.ijpharm.2019.118642, PMID: 31446024

[18] Li Z Liu N Zhang W Wu C Jiang Y Ma J. Integrated transcriptome and proteome analysis provides insight into chilling-induced dormancy breaking in Chimonanthus praecox. Hortic Res. (2020) 7:198. doi: 10.1038/s41438-020-00421-x, PMID: 33328461 PMC7704649

[19] MacLean B Tomazela DM Shulman N Chambers M Finney GL Frewen B. Skyline: an open source document editor for creating and analyzing targeted proteomics experiments. Bioinformatics. (2010) 26:966–8. doi: 10.1093/bioinformatics/btq054, PMID: 20147306 PMC2844992

[20] Gat U DasGupta R Degenstein L Fuchs E. *De novo* hair follicle morphogenesis and hair tumors in mice expressing a truncated Beta-catenin in skin. Cell. (1998) 95:605–14. doi: 10.1016/s0092-8674(00)81631-1, PMID: 9845363

[21] Su R Gong G Zhang L Yan X Wang F Zhang L. Screening the key genes of hair follicle growth cycle in inner Mongolian cashmere goat based on RNA sequencing. Arch Anim Breed. (2020) 63:155–64. doi: 10.5194/aab-63-155-2020, PMID: 32490151 PMC7256851

[22] Krejci P Pejchalova K Rosenbloom BE Rosenfelt FP Tran EL Laurell H. The antiapoptotic protein Api5 and its partner, high molecular weight FGF2, are up-regulated in B cell chronic lymphoid leukemia. J Leukoc Biol. (2007) 82:1363–4. doi: 10.1189/jlb.0607425, PMID: 17827341

[23] Tewari M Yu M Ross B Dean C Giordano A Rubin R. AAC-11, a novel cDNA that inhibits apoptosis after growth factor withdrawal. Cancer Res. (1997) 57:4063–9. PMID: 9307294

[24] Sasaki H Moriyama S Yukiue H Kobayashi Y Nakashima Y Kaji M. Expression of the antiapoptosis gene, AAC-11, as a prognosis marker in non-small cell lung cancer. Lung Cancer. (2001) 34:53–7. doi: 10.1016/s0169-5002(01)00213-6, PMID: 11557113

[25] Kim JW Cho HS Kim JH Hur SY Kim TE Lee JM. Aac-11 overexpression induces invasion and protects cervical cancer cells from apoptosis. Lab Invest. (2000) 80:587–94. doi: 10.1038/labinvest.3780063, PMID: 10780674

[26] Van den Berghe L Laurell H Huez I Zanibellato C Prats H Bugler B. Fif [fibroblast growth factor-2 (FGF-2)-interacting-factor], a nuclear putatively antiapoptotic factor, interacts specifically with Fgf-2. Mol Endocrinol. (2000) 14:1709–24. doi: 10.1210/mend.14.11.0556, PMID: 11075807

[27] Wier EM Neighoff J Sun X Fu K Wan F. Identification of an N-terminal truncation of the NF-Κb P65 subunit that specifically modulates ribosomal protein S3-dependent NF-Κb gene expression. J Biol Chem. (2012) 287:43019–29. doi: 10.1074/jbc.M112.388694, PMID: 23115242 PMC3522296

[28] Seong KM Jung SO Kim HD Kim HJ Jung YJ Choi SY. Yeast ribosomal protein S3 possesses a Β-Lyase activity on damaged DNA. FEBS Lett. (2012) 586:356–61. doi: 10.1016/j.febslet.2011.12.030, PMID: 22245673

[29] Pan R Zhu M Yu C Lv J Guo Y Bian Z. Cancer incidence and mortality: a cohort study in China, 2008-2013. Int J Cancer. (2017) 141:1315–23. doi: 10.1002/ijc.30825, PMID: 28593646

